# Ginsenoside Rg3 Promotes Healthy Aging Through Phosphoinositide‐Associated Lysosomal Regulation in *Caenorhabditis elegans*

**DOI:** 10.1111/acel.70755

**Published:** 2026-10-10

**Authors:** Haiyang Yu, Shiming Li, Yongsheng Chen, Yaoyong Zeng, Junbu Li, Yuxin Guo, Wanxiang Zhang, Lizhu Zhang, Longnong You, Kuncan Chen, Jinhong Wu, Dan Gan, Jiane‐Kang Liu, Lingjun Zheng

**Affiliations:** ^1^ Department of Food Science and Engineering, School of Agriculture and Biology Shanghai Jiao Tong University Shanghai China; ^2^ College of Food Science Fujian Agriculture and Forestry University Fuzhou China; ^3^ Sirio Pharma Co., Ltd Shantou China; ^4^ Department of Neurosurgery Jinshan Hospital of Fudan University Shanghai China; ^5^ Key Laboratory of Systems Biomedicine (Ministry of Education), Shanghai Center for Systems Biomedicine Shanghai Jiao Tong University Shanghai China

**Keywords:** autophagy, *Caenorhabditis elegans*, ginsenoside Rg3, healthy aging, lysosome, phosphoinositides, TFEB, TORC1

## Abstract

Lysosomal decline and proteostasis collapse are defining features of aging and major contributors to neurodegenerative vulnerability, yet interventions that preserve neuronal lysosomal function across aging remain poorly defined. Here, we identify 20(S)‐ginsenoside Rg3, a ginseng‐derived natural product, as a geroprotective small molecule in *Caenorhabditis elegans* . Rg3 extended lifespan and preserved key physiological outputs, including locomotion and pharyngeal pumping, indicating that Rg3 delays functional aging. Rg3 also maintained mechanosensory neuron integrity and touch sensitivity, suppressed neuronal polyglutamine aggregation, and protected dopaminergic neurons against α‐synuclein‐induced degeneration. At the cellular level, Rg3 restored age‐impaired lysosomal function and autophagic flux in neurons. Mechanistically, Rg3 shifted neuronal phosphoinositide reporter signatures toward a PI(4)P‐enriched and PI(3)P‐reduced state, consistent with signaling lipid remodeling at lysosomal membranes. This phosphoinositide shift coincided with reduced RSKS‐1/S6K phosphorylation, indicating suppression of TOR complex 1 (TORC1), and promoted nuclear localization of HLH‐30, the *C. elegans* ortholog of transcription factor EB (TFEB), thereby engaging a conserved lysosomal program. Collectively, these results define Rg3 as a natural‐product geroprotector that couples lysosomal lipid remodeling to TORC1–HLH‐30 signaling to preserve lysosomal competence, neuronal proteostasis, and organismal fitness during aging. Our study nominates phosphoinositide control of lysosomal identity as a therapeutic entry point for healthy aging and neuroprotection.

## Graphical abstract

Ginsenoside Rg3 shifts neuronal phosphoinositide reporter signatures toward increased PI(4)P and reduced PI(3)P signals. These changes are coupled to TORC1 suppression and HLH‐30/TFEB activation, alongside restored autophagic flux and lysosomal competence, preserved neuronal proteostasis, and extended lifespan in *Caenorhabditis elegans* .

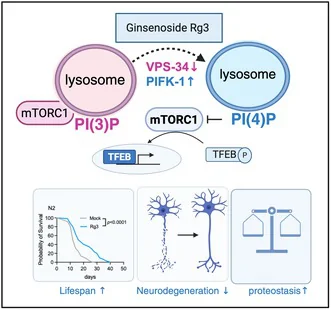

## 1 Introduction

Aging is the dominant risk factor for many neurodegenerative disorders and is accompanied by a progressive decline in cellular quality‐control systems. Long‐lived neurons are particularly vulnerable because damaged organelles and aggregation‐prone proteins cannot be diluted through cell division (Reeve et al. 2014). Declining autophagy–lysosome function therefore provides a mechanistic link between normal aging and the accumulation of amyloid‐β, tau, α‐synuclein, and other proteotoxic species (Nixon and Rubinsztein 2024; Nixon 2013). Interventions that preserve degradative capacity while avoiding broad impairment of essential anabolic functions could therefore promote healthy aging and reduce neuronal vulnerability.

Lysosomes coordinate metabolism, stress responses, and homeostasis as both degradative organelles and signaling platforms (Ballabio and Bonifacino 2020). Mechanistic target of rapamycin complex 1 (mTORC1) is recruited to and activated at the lysosomal surface through the Rag–Ragulator and V‐ATPase‐dependent nutrient sensing (Zoncu et al. 2011). Active mTORC1 promotes TFEB phosphorylation and cytoplasmic retention, whereas reduced mTORC1 signaling permits TFEB nuclear accumulation and induction of lysosomal and autophagy‐related genes (Settembre et al. 2012, 2013). In *C. elegans* , HLH‐30 coordinates autophagy–lysosome gene expression and contributes to longevity (Lapierre et al. 2013). Autophagic activity declines with age, including in neurons (Chang et al. 2017), but how small molecules preserve neuronal lysosomal competence remains incompletely understood.

Endolysosomal signaling is also shaped by membrane phosphoinositides, which act as spatially restricted determinants of organelle identity and recruit trafficking and signaling effectors (Di Paolo and De Camilli 2006). PI(3)P is a defining signal of endosomal membranes and is specifically recognized by FYVE‐domain proteins (Burd and Emr 1998). PI(3)P and PI(4)P are particularly important lipid cues within the endosomal–lysosomal system, but whether pharmacological modulation of these phosphoinositide states can be harnessed to maintain neuronal proteostasis during organismal aging remains unclear.

Ginseng‐derived ginsenosides have anti‐inflammatory, metabolic, and neuroprotective activities (Kim et al. 2017; Wang et al. 2022). Among them, 20(S)‐ginsenoside Rg3 has been linked to neuronal protection in disease models, yet its impact on organismal aging and the underlying mechanisms remain unresolved (Wang et al. 2022).

Here, we show that Rg3 extends lifespan, including when treatment begins during adulthood, and preserves neuronal function in *C. elegans* . Rg3 restores neuronal lysosomal activity and autophagic flux, protects against proteotoxic stress, and shifts phosphoinositide reporter behavior toward a PI(4)P‐enriched and PI(3)P‐reduced state. Genetic and biochemical analyses further associate these effects with reduced TORC1–RSKS‐1 signaling and HLH‐30 activation.

## 2 Materials and Methods

### 2.1 *C. elegans* Strains and Maintenance

Worms were maintained at 20°C on nematode growth medium (NGM) agar plates, with Escherichia coli OP50 provided as the food source, following standard procedures unless otherwise specified (Brenner 1974). Age‐synchronized worm populations for aging‐related assays were obtained by treating gravid adults with hypochlorite. L4‐stage animals were then selected for transfer to NGM medium supplemented with 5‐fluoro‐2′‐deoxyuridine (FUDR) to inhibit progeny production. FUDR was used at a final concentration of 50 mg/L. Complete strain identifiers, genotypes, sources, and construction details are listed in Table S1.

### 2.2 Compound Preparation and Treatment

A 10 mg mL^−1^ stock solution of 20(S)‐ginsenoside Rg3 was prepared in dimethyl sulfoxide (DMSO), diluted with water, and subsequently mixed with concentrated OP50 at a 1:1 ratio. Unless otherwise indicated, worms were treated from the embryonic stage with Rg3 at a final concentration of 10 μg mL^−1^. Mock‐treated controls received the same final concentration of DMSO, which was maintained at 0.1% in all conditions. Dose–response and treatment‐window experiments used the concentrations and start times shown in Figure 1. The Rh2 concentration was selected to approximate the molar concentration of Rg3 and was prepared in the same vehicle.

**FIGURE 1:**
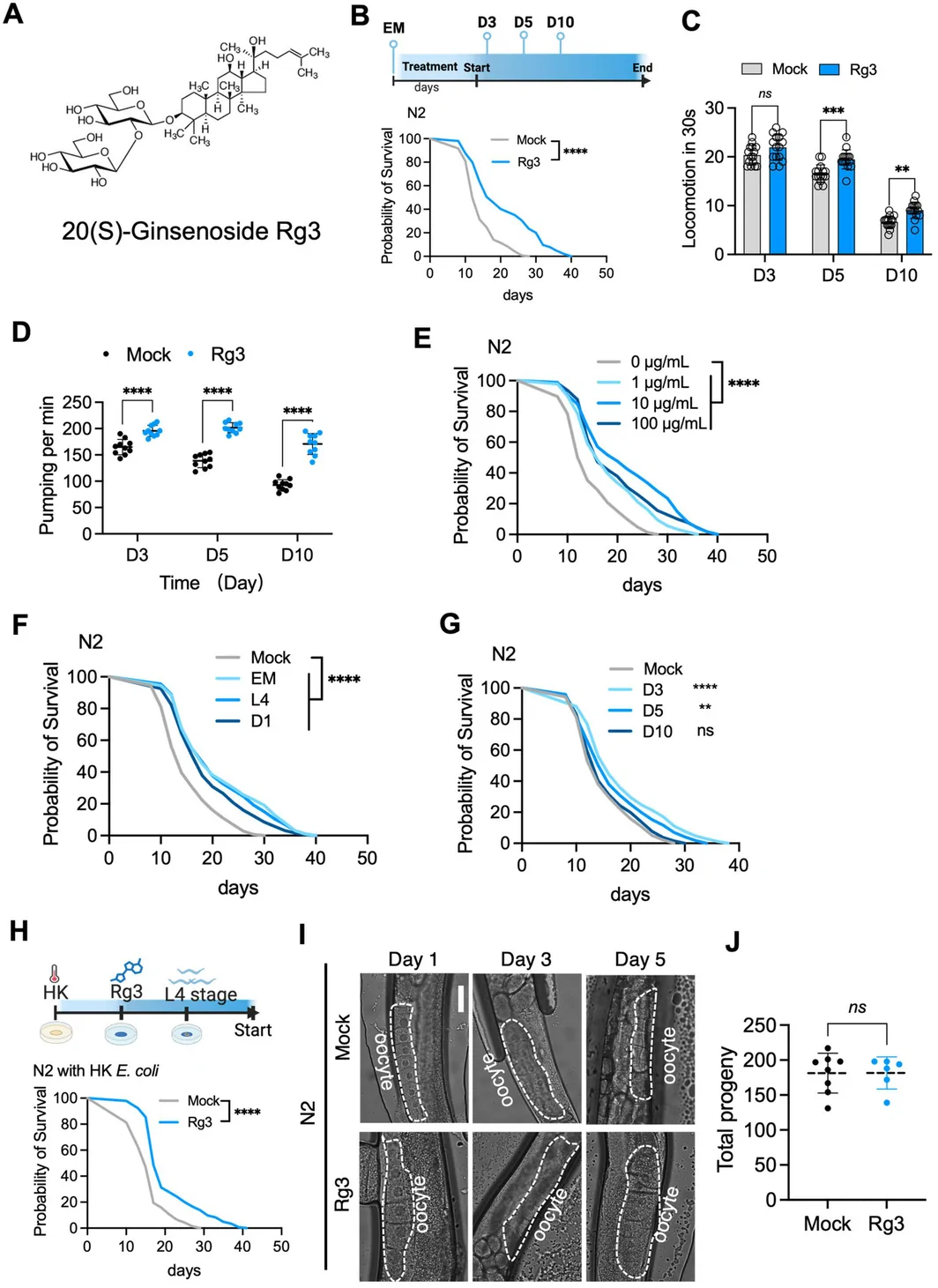
Rg3 extends lifespan and preserves physiological function during aging in *Caenorhabditis elegans* . (A) Chemical structure of 20(S)‐ginsenoside Rg3. (B) Survival curves of wild‐type N2 animals treated with vehicle (Mock) or Rg3. Rg3 was administered at 10 μg mL^−1^. (C) Locomotor activity, measured as body bends per 30 s, in vehicle‐ and Rg3‐treated animals at the indicated adult ages. (D) Pharyngeal pumping rates in vehicle‐ and Rg3‐treated animals at the indicated adult ages. (E) Dose–response analysis of Rg3‐mediated lifespan extension. A concentration of 10 μg mL^−1^ was selected for subsequent experiments. (F) Survival curves of animals exposed to Rg3 beginning at the embryonic, L4, or Day‐1 adult stage. (G) Survival curves of animals exposed to Rg3 beginning on adult Days 3, 5, or 10. (H) Survival curves of animals maintained on heat‐killed *Escherichia coli* OP50 and treated with vehicle or Rg3. (I) Representative differential interference contrast images of oocytes from vehicle‐ and Rg3‐treated animals at the indicated adult ages. Scale bar, 20 μm. (J) Total viable progeny produced per hermaphrodite following vehicle or Rg3 treatment. For (C, D, and J), data are mean ± SD; each point represents one animal, and data were obtained from three independent experiments. Statistical significance was assessed using two‐way ANOVA with Šídák's multiple‐comparison test for C and D; unpaired two‐tailed *t*‐test for J. Survival curves were compared using the Mantel–Cox log‐rank test. **p < 0.01, ***p < 0.001, ****p < 0.0001; ns, not significant.

### 2.3 Lifespan Assays

Lifespan experiments were performed on NGM plates supplemented with FUDR at 20°C, except for temperature‐sensitive *glp‐1*(e2144) animals, which were maintained at 15°C during development and 25°C during adulthood. At least 50 animals were assigned to each genotype and treatment group. Death was recorded when animals showed no response to gentle touch with a platinum wire pick. Animals lost from the plate, killed by internal hatching, or showing vulval rupture were censored. Survival was assessed daily, and experiments were repeated independently at least three times (Kenyon et al. 1993). Heat‐killed OP50 was prepared before seeding.

### 2.4 Locomotion and Pharyngeal Pumping

Locomotor activity was assessed by transferring individual animals to NGM plates and counting body bends over a 30‐s interval following a brief acclimation period. Pharyngeal pumping was assessed by recording contractions in individual animals over a 15‐s interval and expressing the counts as pumps per minute. Each treatment group included at least 15 animals per time point, and all assessments were performed by observers blinded to treatment allocation.

### 2.5 Neuronal Morphology and Behavioral Assays

PVD neuronal morphology was examined using *xrIs37* [*PF49H12.4p::GFP; mec‐3p::mCherry*], and ALM/PLM touch receptor neurons were visualized using *zdIs5* [*mec‐4p::GFP* + *lin‐15*(*+*)]. GABAergic neurons were analyzed using *oxIs12* [*unc‐47p::GFP* + *lin‐15*(*+*)], and AWC chemosensory neurons were examined using *kyIs140* [*str‐2p::GFP* + *lin‐15*(*+*)]. PVD, GABAergic, and AWC neuronal morphology was imaged and quantified at 40× magnification using a Nikon AX microscope. ALM and PLM neuronal morphology were analyzed at 40× magnification using a Zeiss Imager A1 microscope.

Age‐associated PVD degeneration was identified by the appearance of bubble‐like or bead‐like structures along PVD dendrites, as previously described (Lezi et al. 2018). In aged animals, ALM neurons will develop a posteriorly elongated deformation of the cytosol and PLM neurons will show waves and sharp turns in the anterior axon (Wu et al. 2022). In aged animals, the dendrites of GABA neurons throughout the body break down or even disappear. Degenerated AWC neurons will develop bubble‐like vesicular structures and may lead to the disappearance of entire neurons. PVD dendrites and ALM/PLM or AWC processes were assigned to predefined well‐organized, moderately disorganized, or severely disorganized categories by an observer blinded to treatment.

### 2.6 Food Preference Experiments

Food preference was assessed in synchronized N2 animals. Two circles were marked on opposite sides of a 90‐mm NGM plate and seeded with OP50 supplemented with vehicle or Rg3. After the lawns had dried, L4‐stage animals were placed midway between the circles. Animals on each lawn were counted after 3 h.

### 2.7 Oocyte Observation and Progeny Counts

Synchronized hermaphrodites were transferred to 35‐mm NGM plates at the L4 stage. Differential interference contrast (DIC) images were acquired on adult Days 1, 3, 5, and 10 using a Zeiss Imager A1 microscope at 40× magnification. For progeny assays, individual L4 hermaphrodites were transferred to fresh plates every 24 h. Progeny on the previous plate were counted approximately 24 h after transfer. Counting continued until no progeny had been produced for 48 h (Cota et al. 2022).

### 2.8 Chemotaxis Assays

Synchronized animals were maintained on compound‐treated, FUDR‐containing medium until adult Day 3, 5, or 10. Two circles with a radius of 5 mm were marked on opposite sides of a 90‐mm NGM plate and labeled A and B. Circle A received 2 μL of anhydrous ethanol, and circle B received 2 μL of odorant dissolved in anhydrous ethanol. Odorants were 0.1% benzaldehyde or 1% isopentanol (Ferkey et al. 2021). Sodium azide (25 mM) was added to both circles to immobilize arriving animals. Animals were placed midway between the circles, and those within each circle were counted after 1 h.

Chemotaxis Index (CI) = (*N*_B_−*N*_A_)/(*N*_B_ + *N*_A_).

### 2.9 Quantification of Tandem mCherry–GFP–LGG‐1 Puncta

MAH508 sqEx67 [*rgef‐1p::mCherry::GFP::lgg‐1* + *rol‐6*] animals were imaged on Days 3, 5, and 10 of adulthood using Nikon AX Confocal Microscope System (Nikon Corporation, Tokyo, Japan). Z‐stacks were acquired under identical settings and converted to maximum‐intensity projections. Fluorescence analysis was performed within a single, manually delineated region of interest (ROI) encompassing the head of each animal, from the nose tip to the posterior boundary of the terminal pharyngeal bulb. Tandem mCherry–GFP–LGG‐1 puncta were counted in ImageJ after excluding elongated or linear fluorescent structures. The numbers of mCherry‐positive/GFP‐negative and mCherry‐positive/GFP‐positive puncta were recorded separately for each animal. The red‐only fraction was calculated as mCherry‐only puncta divided by the sum of mCherry‐only and double‐positive puncta, and the background‐corrected integrated mCherry/GFP fluorescence intensity ratio was calculated for each animal.

### 2.10 Fluorescence Imaging and Quantification of PI(3)P and PI(4)P

Age‐synchronized ZHY261 (*lswIs85* [*unc‐119p::GFP::2* × *FYVE* + *sur‐5p::RFP*]) and ZHY168 (*unc‐76*(*e911*) *V; IswEx107* [*unc‐119p::GFP::OSBP*(*PH*) + *unc‐76*(*+*)]) were maintained under Mock or Rg3‐treated conditions and imaged on adult Days 3, 5, and 10. Imaging was performed with a 100× objective on an Olympus FV3000 laser‐scanning confocal microscope (Olympus, Tokyo, Japan). Images were acquired under identical settings within each experiment. For each animal, GFP‐positive puncta in the nerve ring region, defined as the area between the metacorpus and the terminal pharyngeal bulb, were quantified using ImageJ (NIH, USA). GFP::OSBP(PH)‐positive puncta were quantified as PI(4)P‐positive structures, whereas GFP::2 × FYVE‐positive puncta were quantified as PI(3)P‐positive structures following background subtraction and thresholding using identical parameters across groups. Each plotted point corresponds to a single animal (*n* > 15).

### 2.11 LysoTracker Red Staining and Fluorescence Colocalization Analysis

On Day 10 of adulthood, mock‐ and Rg3‐treated ZHY168 (*unc‐76*(*e911*) *V; IswEx107* [*unc‐119p::GFP::OSBP*(*PH*) + *unc‐76*(*+*)]) animals were stained with 10 μM LysoTracker Red DND‐99 (Invitrogen, Thermo Fisher Scientific, L7528) at room temperature for 1 h in the dark. Following staining, animals were placed on fresh NGM plates seeded with OP50 for a 30‐min recovery period prior to imaging. Fluorescence imaging was performed on an Olympus FV3000 laser‐scanning confocal microscope with a 100× objective, using the same acquisition parameters for all groups. GFP and LysoTracker Red fluorescence were acquired in two channels using 488‐ and 561‐nm laser excitation, respectively. For line‐scan analysis, corresponding GFP and LysoTracker Red images were merged in ImageJ. A straight‐line ROI was drawn across representative fluorescent structures, and fluorescence intensity profiles of both channels were obtained using the Plot Profile function and plotted against distance along the indicated line to visualize their spatial colocalization.

### 2.12 Quantification of HLH‐30 Nuclear Localization

Day‐5 MAH240 *sqIs17* [*hlh‐30p::hlh‐30::GFP* + *rol‐6*(*su1006*)*]* adults treated with vehicle or Rg3 were imaged in the intestinal region using Nikon AX Confocal Microscope System (Nikon Corporation, Tokyo, Japan) with a × 20 objective. The number of intestinal nuclei showing HLH‐30::GFP accumulation was counted for each animal in ImageJ.

### 2.13 Reverse‐Transcription Quantitative PCR

Total RNA was extracted from age‐matched animals subjected to the indicated RNAi and compound treatment conditions using an RNA isolation reagent. RNA samples were evaluated for concentration and purity prior to reverse transcription. Equal amounts of total RNA were used for cDNA synthesis with a Vazyme Biotech Co. Ltd. reverse transcription kit. Quantitative PCR was carried out using Vazyme qPCR reagents on a Bio‐Rad real‐time PCR platform.

The expression levels of *let‐363, daf‐15, rict‐1, pifk‐1, atg‐9, atg‐18, lgg‐1, vha‐16, vha‐15, vha‐18, lmp‐1, ctsa‐1.1, cpr‐3*, and *asp‐1* were quantified. Relative mRNA expression was normalized to *cdc‐42* as the internal reference gene and calculated using the 2^−ΔΔCt^ method. The primer sequences used in this study are listed in Table S2. All experiments included independent biological replicates, with each qPCR reaction additionally run in technical replicates. Data were analyzed according to the procedures outlined in the Statistical analysis section.

### 2.14 Western Blotting

Age‐synchronized *C. elegans* were maintained on NGM plates in the presence or absence of Rg3 until they reached the L4 stage. Animals were harvested, rinsed three times with ice‐cold M9 buffer, and lysed in RIPA buffer containing protease and phosphatase inhibitors (Beyotime, P1045). Proteins were resolved by SDS‐PAGE using gradient polyacrylamide gels (Servicebio, G2193‐50T) and transferred to PVDF membranes (GVS). Membranes were incubated at 4°C overnight with an anti‐phospho‐p70 S6 Kinase (Thr389) antibody (1:500; Cell Signaling Technology, #9205), which was used to detect phosphorylated *C. elegans* RSKS‐1. Membranes were then washed with TBST and probed with HRP‐conjugated goat anti‐rabbit IgG (1:10,000; Yeasen, 33101ES). For α‐tubulin detection, membranes were probed with an anti‐α‐tubulin antibody (1:1000; Sigma, T6074), followed by an HRP‐conjugated goat anti‐mouse IgG (H + L) secondary antibody (1:10,000; Yeasen, 33201ES). Chemiluminescent signals were captured using a GE Amersham Imager 600 (GE Healthcare Life Sciences, Uppsala, Sweden). Phosphorylated RSKS‐1 signals were normalized to the corresponding α‐tubulin signals.

### 2.15 RNA Sequencing

Total RNA was extracted from age‐matched N2 animals treated with vehicle or Rg3, with three independent biological replicates prepared for each group. RNA isolation, quality control, library construction, transcriptome sequencing, and initial bioinformatic analyses were performed by Majorbio Bio‐pharm Technology Co. Ltd. (Shanghai, China). RNA concentration, purity, and integrity were evaluated prior to library preparation, and qualified RNA samples were used to generate sequencing libraries.

Paired‐end sequencing (150 bp) was conducted on an Illumina NovaSeq 6000 platform. Raw sequencing data were filtered to remove adapter sequences, low‐quality reads, and reads containing ambiguous bases. Clean reads were subsequently mapped to the *Caenorhabditis elegans* reference genome (WBcel235), followed by gene‐level expression quantification and normalization using standard RNA‐seq procedures.

Differential expression analysis comparing vehicle‐ and Rg3‐treated animals was conducted with DESeq2 on the Majorbio Cloud Platform. Multiple‐testing correction was applied to the resulting *p* values to control the false discovery rate. Differentially expressed genes were identified using thresholds of adjusted *p* < 0.05 and absolute log_2_ fold change ≥ 1. These genes were subsequently subjected to functional annotation and enrichment analyses using Gene Ontology and Kyoto Encyclopedia of Genes and Genomes pathways to identify biological processes and pathways associated with Rg3 treatment.

### 2.16 Lysosome Morphology and Acidity

Adult *C. elegans* expressing NUC‐1::mCherry were examined by laser‐scanning confocal microscopy on Days 3, 5, and 10 of adulthood. ImageJ was used to determine the lengths of lysosomal tubules labeled with NUC‐1::mCherry in individual animals. Tubules that crossed one another were treated as separate structures during quantification. Five tubules were measured in each animal, with at least 15 animals assessed per strain at each time point (Yanan Sun et al. 2020).

To assess lysosomal acidity, approximately 40 adult worms from each age group were incubated in 80 μL of M9 buffer containing 100 μM LysoSensor Green DND‐189 and LysoTracker Red (Thermo Fisher) for intestinal and subcutaneous staining. Animals were stained for 1 h at 20°C in the dark and subsequently transferred to NGM plates seeded with fresh OP50. After a further 1‐h recovery period under the same temperature and light conditions, worms were examined, and the relative LSG/LTR fluorescence intensity was quantified using ImageJ (Sun et al. 2020).

### 2.17 RNAi Treatment

Feeding‐mediated RNAi was performed using bacteria carrying either a target‐specific RNAi construct or the L4440 empty vector as a control. To prepare RNAi plates, autoclaved agar medium was supplemented with carbenicillin (100 μg/mL) and isopropyl β‐D‐1‐thiogalactopyranoside (IPTG; 1 mM). Unless indicated otherwise, synchronized animals were maintained on these plates from hatching onward. Bacterial RNAi clones were sourced from the Ahringer RNAi library and used to target *bec‐1*, *vps‐34*, *vha‐3*, *pifk‐1*, *hlh‐30*, *let‐363*, and *rsks‐1*, with an additional condition targeting *pifk‐1* and *let‐363* simultaneously. Details of the RNAi constructs are provided in Table S3.

### 2.18 Neuronal Proteotoxicity Assays

Analysis of neurodegenerative lesions in worms was performed at specified time points (Day 3, Day 5, and Day 10 post‐adulthood) (Harrington et al. 2010). On the day of assessment, 15–25 randomly selected worms were mounted on a glass slide in 20 μL of 25 mM levamisole for immobilization. The head region of each animal was then examined and imaged using a fluorescence microscope.

For UA44, we primarily assessed whether head neurons were lost. Healthy worm heads have six intact dopamine neurons, including a pair of dorsal CEPs, a pair of ventral CEPs, and a pair of ADEs. Here, we counted the number of neurons present in each observed worm (Vijayan et al. 2019). For AM101, we counted the number of Poly Q aggregates in the head neural ring neurons.

### 2.19 Statistical Analysis

Analyses were performed in GraphPad Prism 10.3.0. Survival was compared by log‐rank testing. Continuous outcomes were analyzed by two‐tailed tests or one‐ or two‐way analysis of variance with multiplicity‐adjusted post hoc comparisons. Ordinal neuronal categories were analyzed by ordinal or multinomial models, or by contingency tests when only category counts were available. The animal, not the image, field, punctum, or organelle, was treated as the experimental unit unless a hierarchical model was used. Unless otherwise indicated, results are presented as the mean ± standard deviation (SD). Exact *p* values are provided whenever possible, with statistical significance denoted as follows: *p < 0.05, **p < 0.01, ***p < 0.001, and ****p < 0.0001. The corresponding figure legends specify sample sizes, biological replicates, exclusions, and the statistical test applied to each panel.

## 3 Results

### 3.1 Rg3 Extends Lifespan and Preserves Physiological Function During Aging

We investigated the low‐abundance saponin 20(S)‐ginsenoside Rg3 (Figure 1A), which has reported neuroprotective activities (Liu et al. 2024) (Qi et al. 2024). Rg3 supplementation significantly extended lifespan in wild‐type N2 animals (Figure 1B).

Rg3 also preserved locomotor activity and pharyngeal pumping in aged adults (Figure 1C,D), two healthspan measures that decline with age (Ryu et al. 2016). Dose–response analysis identified 10 μg mL^−1^ as an effective concentration under the conditions tested (Figure 1E).

Rg3 extended lifespan when treatment began at the embryonic, L4, or Day‐1 adult stage (Figure 1F). Treatment initiated on adult Day 3 or 5 also conferred a lifespan benefit, whereas initiation on adult Day 10 did not (Figure 1G). Thus, developmental exposure was not required, although the detectable benefit depended on treatment timing.

Rh2, a metabolite of Rg3 in mammalian systems, did not extend lifespan under the conditions tested (Figure S1A,B), consistent with previous findings in worms (Wang et al. 2021). Rh2 therefore did not phenocopy the longevity benefit of Rg3.

We next examined potential feeding‐related confounders. Rg3 did not alter OP50 growth (Figure S1C), and its lifespan benefit persisted with heat‐killed OP50 (Figure 1H). Animals also showed no avoidance of Rg3‐supplemented lawns in a two‐choice assay (Figure S1D). These findings argue against an explanation based solely on bacterial growth inhibition or food avoidance.

Rg3 did not significantly alter progeny production (Figure 1I,J), indicating that lifespan extension was not accompanied by reduced fecundity under these conditions.

Collectively, these data show that ginsenoside Rg3 extends lifespan while maintaining late‐life physiological performance through mechanisms independent of dietary restriction and fertility reduction, establishing Rg3 as a bona fide small‐molecule geroprotective candidate in *C. elegans* .

### 3.2 Rg3‐Mediated Lifespan Extension Depends on Autophagy–Lysosome Function and Involves TORC1–RSKS‐1 Signaling

To investigate longevity pathways associated with the Rg3 response, we tested several genetic backgrounds. Rg3 extended lifespan in *daf‐16*(*mu86*) mutants (Lin et al. 1997), indicating that DAF‐16/FOXO was dispensable under these conditions (Figure 2A). The response also persisted in *eat‐2*(*ad1116*), a dietary restriction model (Hansen et al. 2008), and in *aak‐2*(*gt33*) and *clk‐1*(*qm30*) mutants affecting AMPK and mitochondrial function, respectively (Apfeld et al. 2004) (Felkai et al. 1999) (Figure 2C,D). Rg3 further extended lifespan in germline‐defective *glp‐1*(*e2144*) animals (Hsin and Kenyon 1999) (Figure 2E).

**FIGURE 2:**
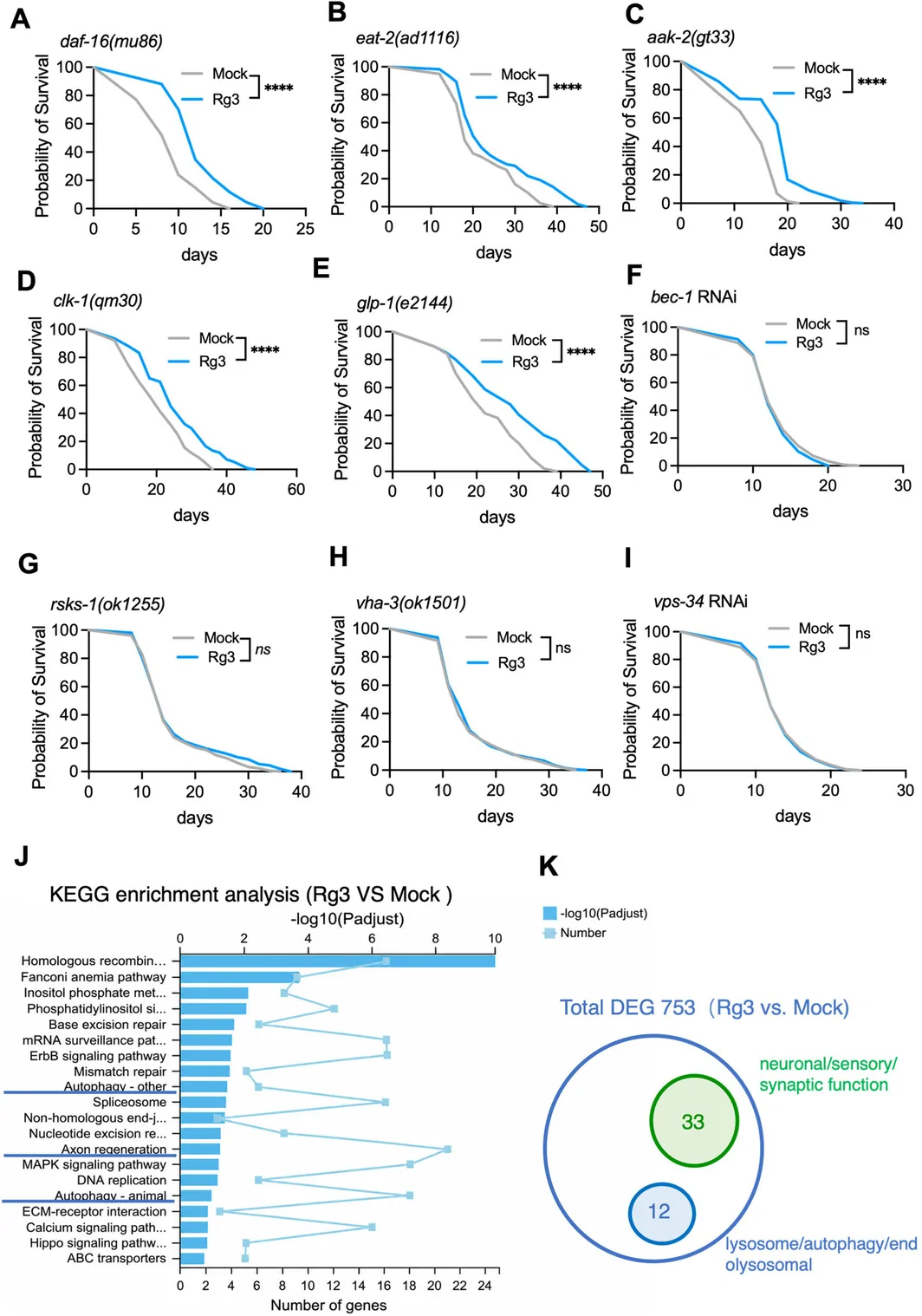
Rg3‐mediated longevity remains detectable in several canonical longevity mutants but is lost following disruption of autophagy–lysosome genes. (A–E) Survival curves of (A) *daf‐16*(*mu86*), (B) *eat‐2*(*ad1116*), (C) *aak‐2*(*gt33*), (D) *clk‐1*(*qm30*), and (E) *glp‐1*(*e2144*) animals treated with vehicle or Rg3. For experiments using the temperature‐sensitive *glp‐1*(*e2144*) allele, animals were maintained at 15°C during development and 25°C during adulthood. (F) Survival curves of animals subjected to control or *bec‐1* RNA interference (RNAi) and treated with vehicle or Rg3. (G) Survival curves of *rsks‐1*(*ok1255*) animals treated with vehicle or Rg3. (H) Survival curves of *vha‐3*(*ok1501*) animals treated with vehicle or Rg3. (I) Survival curves of animals subjected to control or *vps‐34* RNAi and treated with vehicle or Rg3. (J) KEGG enrichment analysis of differentially expressed genes in Rg3‐treated animals relative to vehicle‐treated controls. Differentially expressed genes were defined by |log_2_(fold change)| ≥ 1 and adjusted *p* < 0.05. Gene number and enrichment significance are represented as indicated. (K) Functional classification of Rg3‐regulated genes associated with autophagy–lysosome/endolysosomal function or neuronal, sensory, and synaptic processes. Survival curves were compared using the Mantel–Cox log‐rank test. **** *p* < 0.0001; ns, not significant.

By contrast, *bec‐1* RNAi abolished detectable Rg3‐induced lifespan extension (Figure 2F). BEC‐1 is the Beclin 1/ATG6 ortholog involved in autophagy nucleation (Melendez et al. 2003) (Hansen et al. 2008). Rg3 also failed to further extend lifespan in *rsks‐1*(*ok1255*) mutants lacking the TORC1‐associated S6 kinase ortholog (Figure 2G) (Korta et al. 2012), or in *vha‐3*(*ok1501*) mutants affecting V‐ATPase‐dependent lysosomal function (Figure 2H) (Pu and Qi 2024). Knockdown of vps‐34, which encodes the Class III PI 3‐kinase involved in PI(3)P production and autophagy initiation, likewise prevented lifespan extension (Hansen et al. 2008) (Figure 2I). These findings support a requirement for autophagy–lysosome function and suggest that Rg3 acts through, or converges on, TORC1–RSKS‐1 signaling.

RNA sequencing identified 753 differentially expressed genes following Rg3 treatment. Kyoto Encyclopedia of Genes and Genomes (KEGG) analysis revealed enrichment of phosphatidylinositol signaling, inositol phosphate metabolism, autophagy, and axon regeneration pathways (Figure 2J). Functional annotation identified 12 genes associated with lysosome, autophagy, or endolysosomal processes and 33 associated with neuronal, sensory, or synaptic processes (Figure 2K).

Axon regeneration‐associated genes included regulators of kinase signaling (e.g., *pmk‐1*, *mlck‐1*, and *zak‐1*), TOR signaling (*let‐363*), calcium transport (*nca‐2*), and axon guidance or synaptic function (*unc‐13* and *lin‐12*) (Table S4). Together, these findings associate the Rg3 response with autophagy–lysosome pathways and neuronal maintenance.

### 3.3 Rg3 Preserves Neuronal Morphology, Function, and Proteostasis During Aging

The enrichment of axon‐regeneration and neuronal maintenance pathways among Rg3‐responsive genes prompted us to test whether Rg3 protects neuronal integrity during aging. We first examined the PVD multidendritic sensory neurons, which mediate harsh‐touch responses and develop age‐associated dendritic varicosities (Krishna et al. 2025). Rg3 preserved PVD organization and reduced morphological disorganization at later adult ages (Figure 3A,B). Rg3 also improved touch responsiveness, with a significant benefit on adult Day 10 (Figure 3C), supporting preservation of neuronal morphology and function.

**FIGURE 3:**
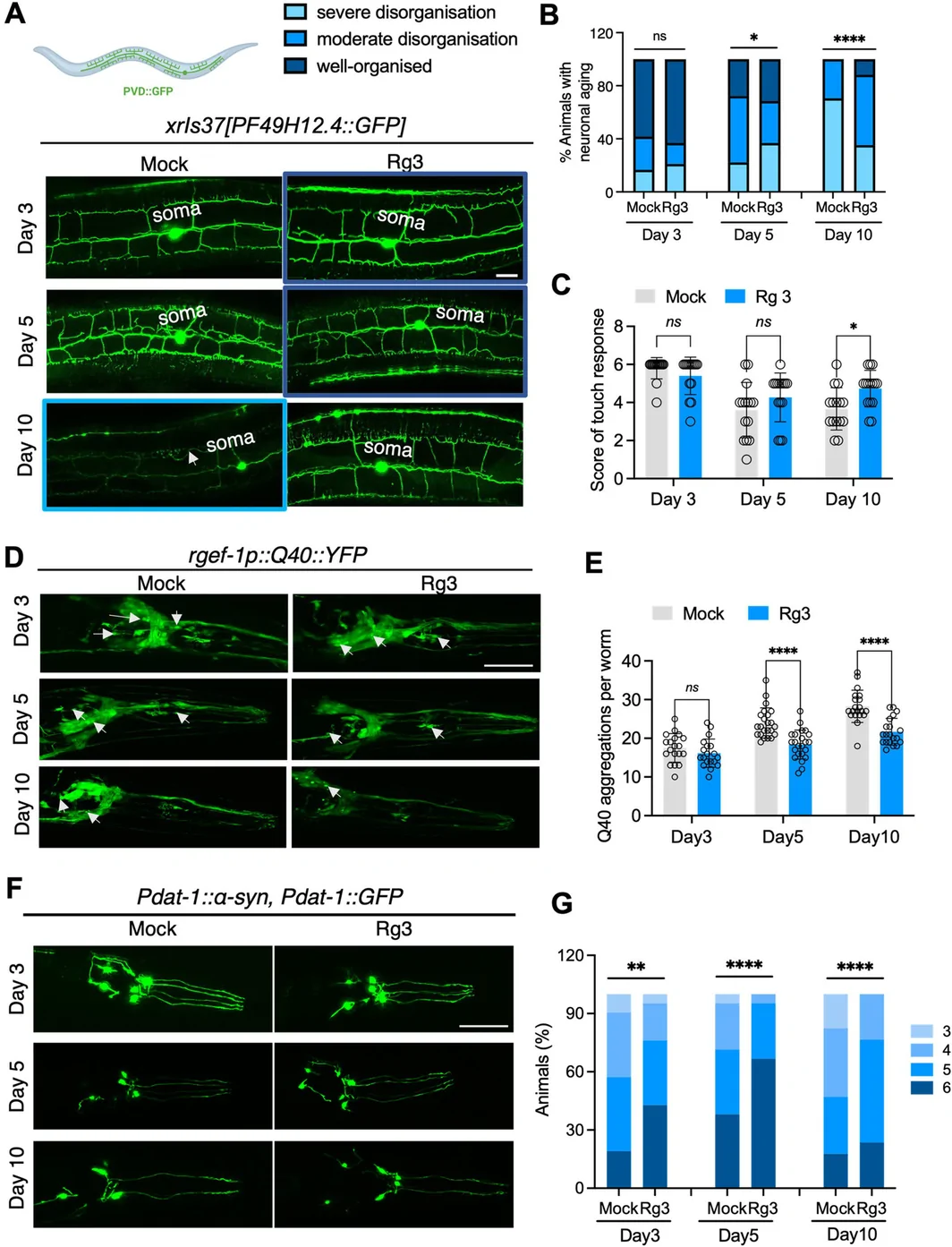
Rg3 preserves neuronal architecture and proteostasis during aging. (A) Representative images of PVD neurons labeled with *xrIs37* [*PF49H12.4p::GFP; Pmec‐3::mCherry]* in vehicle‐ and Rg3‐treated animals on adult Days 3, 5, and 10. Scale bar, 20 μm. (B) Quantification of age‐associated PVD morphological abnormalities. Animals were classified as normal, mildly disorganized, or severely disorganized according to the predefined criteria described in the Materials and methods. Scoring was performed with the investigator blinded to treatment. (C) Number of responses to posterior harsh‐touch stimulation at the indicated adult ages. Each animal was stimulated six times, and the number of trials eliciting a behavioral response was recorded. Behavioral scoring was performed by an investigator blinded to treatment. (D) Representative images of neuronal Q40::YFP aggregation in *rmIs110* [*F25B3.3p::Q40::YFP]* animals treated with vehicle or Rg3 on adult Days 3, 5, and 10. Scale bar, 25 μm. (E) Quantification of neuronal Q40::YFP aggregates per animal. (F) Representative images of dopaminergic neurons in animals expressing human α‐synuclein and GFP under the *dat‐1* promoter following vehicle or Rg3 treatment. Images were acquired on adult Days 3, 5, and 10. Scale bar, 50 μm. (G) Percentages of animals with 3, 4, 5, or 6 dopaminergic (DA) neurons on adult Days 3, 5, and 10 following treatment with Rg3 or mock control. Colors indicate the number of DA neurons per animal. For (C, E), data are mean ± SD. For (B, G), data show the percentages of animals in the indicated categories. Data were obtained from three independent experiments, with *n* ≥ 15 animals per condition. PVD morphology and dopaminergic neuron integrity were analyzed using two‐sided Fisher's exact test comparing Mock and Rg3 groups at each time point. Aggregate numbers and touch responses were analyzed using two‐way ANOVA followed by Šídák's multiple‐comparison test. *p < 0.05, **p < 0.01, ****p < 0.0001; ns, not significant.

We next examined ALM and PLM touch receptor neurons using a *Pmec‐4::GFP* (*zdIs5*) reporter (Ko et al. 2020) (Ghosh‐Roy et al. 2010). Rg3 attenuated age‐associated abnormalities, including ectopic processes, wavy axons, and acute kinks (Figure S2A–C), suggesting broader protection of mechanosensory neuron morphology.

AWC neurons detect volatile odorants and regulate chemotaxis (Leinwand et al. 2015). Using str‐2p::GFP (Sun and Jin 2023), we observed improved AWC morphological organization following Rg3 treatment on adult Days 5 and 10, but no significant difference on Day 3 (Figure S2D,E). Rg3 also increased chemotaxis indices toward isopentanol and benzaldehyde (Figure S2F–H), supporting preservation of chemosensory function.

We then examined neuronal proteotoxicity, which can be modeled in *C. elegans* by expression of aggregation‐prone proteins (Wang et al. 2024). In animals expressing neuronal Q40::YFP (Berendzen et al. 2016), Rg3 reduced aggregate numbers on adult Days 5 and 10 (Figure 3D,E). In an α‐synuclein model, Rg3 increased the proportion of animals retaining more dopaminergic neurons on adult Days 3, 5, and 10 (Figure 3F,G). These findings support a protective effect against neuronal protein aggregation and α‐synuclein‐associated toxicity.

### 3.4 Rg3 Promotes Neuronal Autophagic Flux and Requires VHA‐3 for Protection Against Neuronal Aging

To assess neuronal autophagy during aging, we used the tandem reporter rgef‐1p::mCherry::GFP::*lgg‐1* (Chang et al. 2017). GFP is quenched in acidic compartments, whereas mCherry is retained; double‐positive puncta therefore represent autophagosome‐like compartments, and red‐only puncta report acidic autolysosomal compartments (Chang et al. 2017) (Figure 4A). We quantified puncta and the mCherry‐to‐GFP fluorescence ratio to assess autophagic flux.

**FIGURE 4:**
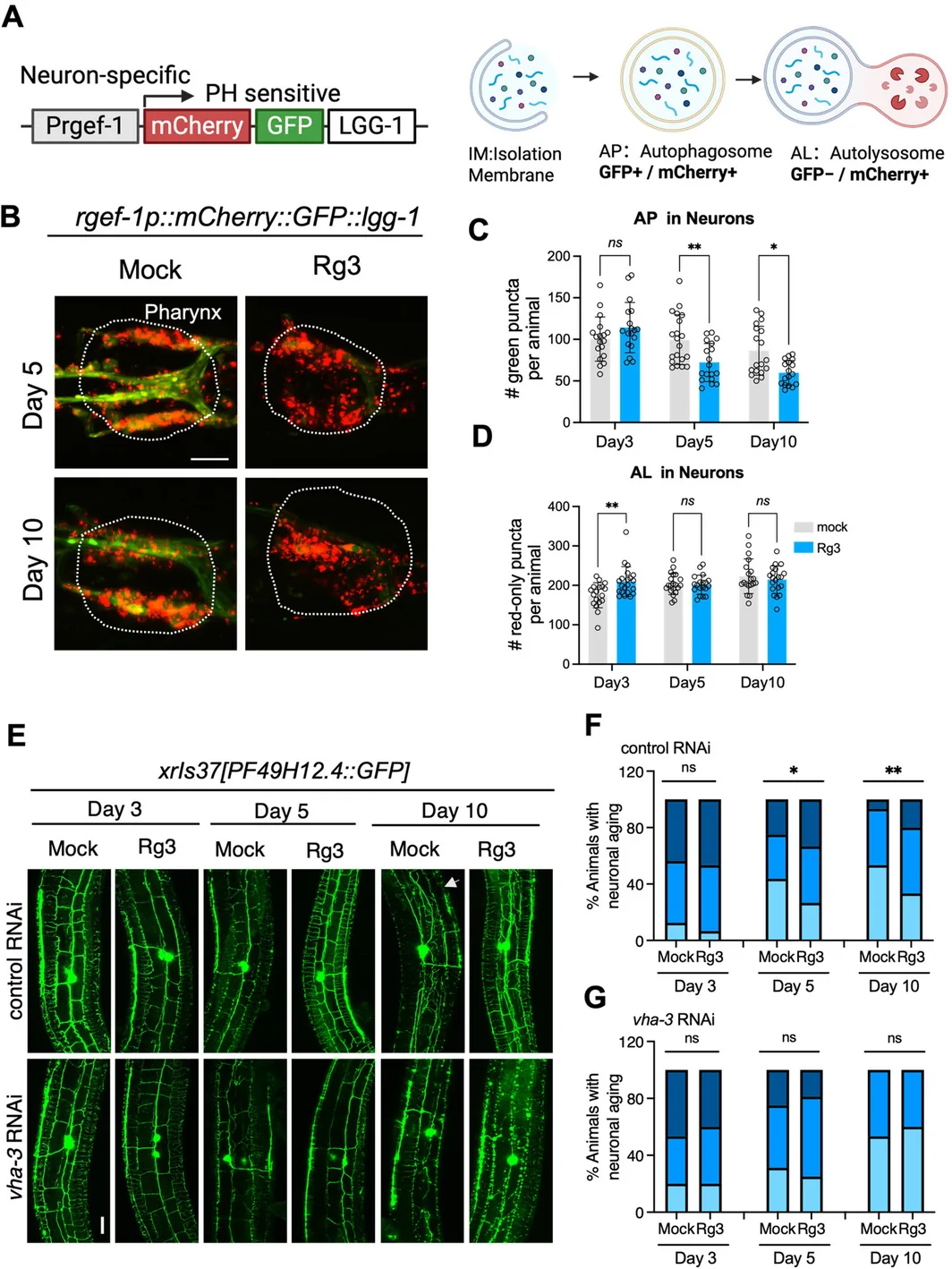
Rg3 promotes the formation of acidic neuronal autolysosomal compartments and requires VHA‐3 for protection against PVD aging. (A) Schematic of the neuron‐specific tandem fluorescent autophagy reporter *rgef‐1p::mCherry::GFP::lgg‐1*. GFP fluorescence is quenched following delivery to acidic lysosomal compartments, whereas mCherry fluorescence is relatively acid resistant. GFP^+^mCherry^+^ puncta are therefore consistent with autophagosomes, whereas GFP^−^mCherry^+^ puncta are consistent with acidic autolysosomal compartments. The diagram was created using BioRender.com. (B) Representative images of neuronal mCherry::GFP::LGG‐1 puncta in vehicle‐ and Rg3‐treated animals on adult days 5 and 10. Scale bar, 10 μm. (C) Number of mCherry^+^GFP^+^ LGG‐1 puncta per animal at the indicated adult ages. (D) Number of mCherry^+^GFP^−^ LGG‐1 puncta per animal at the indicated adult ages. (E) Representative images of PVD neurons labeled with *xrIs37* in animals subjected to control or *vha‐3* RNAi and treated with vehicle or Rg3. Scale bar, 20 μm. (F, G) Quantification of age‐associated PVD morphological abnormalities following (F) control RNAi or (G) *vha‐3* RNAi. For (C, D), data are mean ± SD from three independent experiments, with *n* ≥ 15 animals per condition. Puncta numbers were analyzed using two‐way ANOVA followed by Šídák's multiple‐comparison test. Panels F and G show the percentages of animals in the indicated morphological categories. *p < 0.05, **p < 0.01; ns, not significant.

Rg3 decreased GFP‐positive LGG‐1 puncta on adult Days 5 and 10, but not Day 3 (Figure 4B,C). Red‐only puncta increased on Day 3 but were not significantly altered at later ages (Figure 4D). The mCherry‐to‐GFP ratio increased on Days 5 and 10 (Figure S3A,B). Together, these findings support improved neuronal autophagic flux during aging.

We next tested whether VHA‐3 was required for neuronal protection using the PVD reporter *xrIs37* (Figure 4E). Under control RNAi, Rg3 reduced age‐associated PVD morphological abnormalities on adult Days 5 and 10 (Figure 4F). This benefit was absent following *vha‐3* RNAi (Figure 4G). Because *vha‐3* encodes a V‐ATPase subunit involved in organelle acidification (Syntichaki et al. 2005), these findings support a requirement for VHA‐3 and suggest that lysosomal acidification contributes to Rg3‐mediated neuronal protection.

We also examined lysosomal homeostasis using hypodermal NUC‐1::mCherry (Y. Sun et al. 2020). Rg3 reduced lysosomal tubule length on adult Day 10 and vesicular lysosome accumulation on Days 5 and 10 (Figure S4A–C). Consistent with age‐related changes in lysosomal acidity (Baxi et al. 2017) (Y. Sun et al. 2020), the LysoSensor Green‐to‐LysoTracker Red ratio varied with age and was altered by Rg3 across the ages examined (Figure S4D,E).

Together, these findings support a role for Rg3 in preserving lysosomal homeostasis during aging.

### 3.5 Rg3 Remodels Neuronal Phosphoinositide Homeostasis Through PIFK‐1‐Dependent PI(4)P Signaling

The transcriptomic dataset contained five downregulated genes encoding FYVE‐domain‐containing proteins: *R11D1.10*, *eea‐1*, *rabs‐5*, *aka‐1*, and *wdfy‐3* (Table S5). Because FYVE domains bind PI(3)P (Hu et al. 2023), we examined whether Rg3 altered neuronal phosphoinositide reporter signals.

Using neuronal GFP::2 × FYVE (Hu et al. 2023), we observed an age‐dependent increase in PI(3)P reporter‐positive puncta in controls. Rg3 reduced puncta accumulation on adult Days 5 and 10, but not Day 3 (Figure 5A,B), indicating altered PI(3)P‐associated compartment abundance.

**FIGURE 5:**
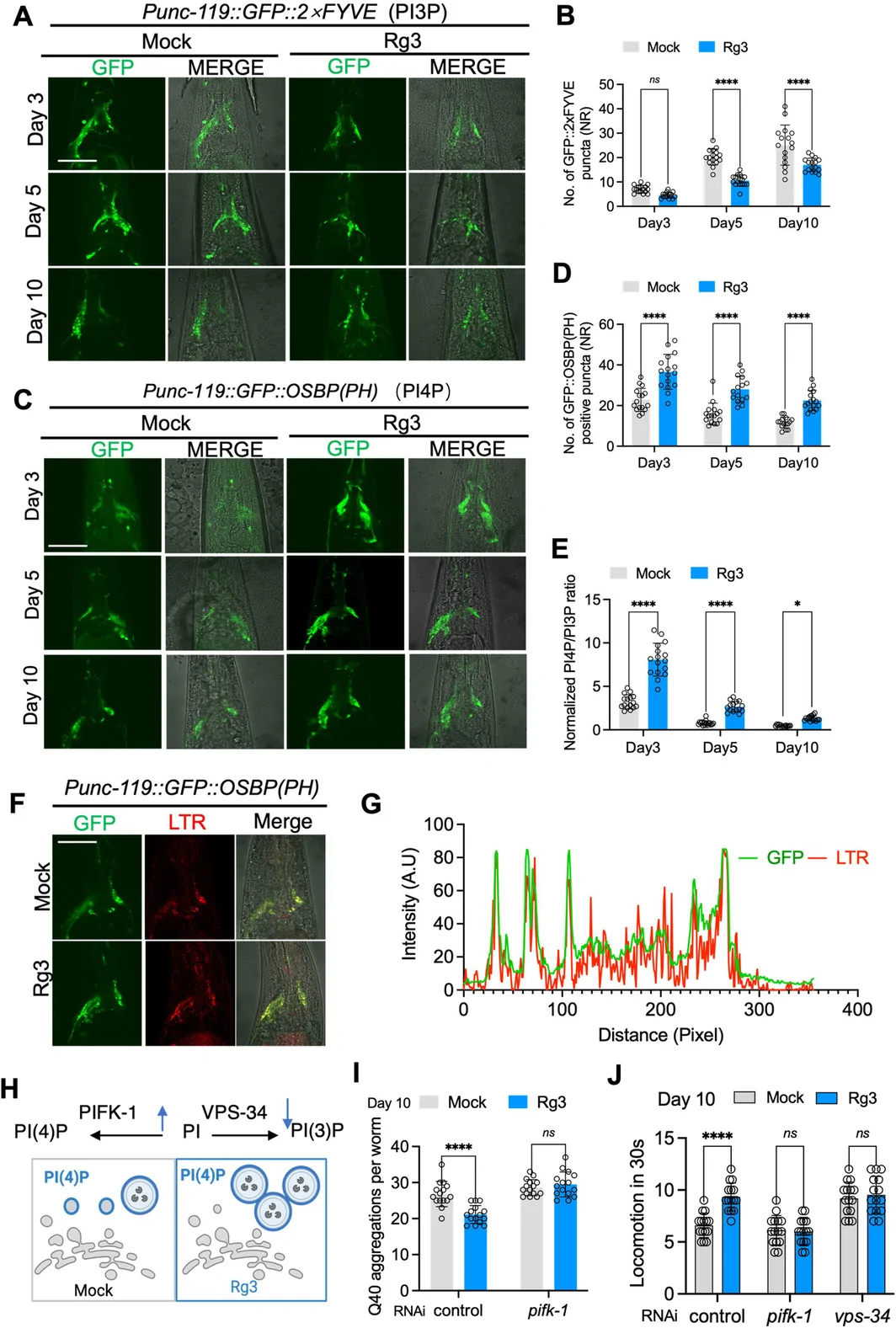
Rg3 shifts neuronal phosphoinositide reporter signals toward PI(4)P, whereas PIFK‐1 is required for its proteostatic benefits. (A) Representative images of neuronal PI(3)P reporter‐positive structures visualized using *Punc‐119::GFP::2 × FYVE* in vehicle‐ and Rg3‐treated animals on adult Days 3, 5 and 10. Scale bar, 20 μm. (B) Quantification of GFP::2 × FYVE‐positive puncta per animal. (C) Representative images of neuronal PI(4)P reporter‐positive structures visualized using *Punc‐119::GFP::OSBP*(*PH*) in vehicle‐ and Rg3‐treated animals at the indicated adult ages. Scale bar, 20 μm. (D) Quantification of GFP::OSBP(PH)‐positive puncta per animal. (E) Relative PI(4)P‐to‐PI(3)P reporter index, calculated from the normalized GFP::OSBP(PH) and GFP::2 × FYVE signals obtained from the corresponding reporter strains. This index represents relative reporter behavior and not the absolute molar ratio of PI(4)P to PI(3)P. (F) Representative images showing colocalization between GFP::OSBP(PH) reporter‐positive structures and LysoTracker Red‐labeled acidic compartments following Rg3 treatment. Scale bar, 20 μm. (G) Representative line‐scan fluorescence intensity profiles of GFP::OSBP(PH) and LysoTracker Red (LTR) signals across the region of interest indicated in (F), showing spatial overlap between PI(4)P reporter‐positive structures and acidic compartments. Fluorescence intensity is expressed in arbitrary units (A.U.). (H) Simplified schematic illustrating the predicted contributions of PIFK‐1 and VPS‐34 to PI(4)P and PI(3)P production, respectively. The diagram was created using BioRender.com. (I) Quantification of neuronal Q40::YFP aggregates in animals subjected to control or *pifk‐1* RNAi and treated with vehicle or Rg3. (J) Locomotor activity in animals subjected to control, *pifk‐1* or *vps‐34* RNAi and treated with vehicle or Rg3. Data are mean ± SD from three independent experiments, with *n* ≥ 15 animals per condition. Data were analyzed using two‐way ANOVA followed by Šídák's multiple‐comparison test. PI(3)P, phosphatidylinositol 3‐phosphate; PI(4)P, phosphatidylinositol 4‐phosphate; PH, pleckstrin homology domain. *p < 0.05, ****p < 0.0001; ns, not significant.

Because phosphoinositide identity is dynamically balanced across lysosomal membranes, we next examined PI(4)P using *Punc‐119::GFP::OSBP*(*PH*) (Hu et al. 2023). In contrast to its effect on PI(3)P, Rg3 significantly increased the number of GFP::OSBP(PH)‐positive puncta at Days 3, 5, and 10 (Figure 5C,D). Consistently, the PI(4)P/PI(3)P reporter ratio was significantly elevated by Rg3 across all examined ages (Figure 5E). These findings suggest that Rg3 shifts neuronal phosphoinositide balance from a PI(3)P‐enriched state toward PI(4)P‐enriched membrane compartments during aging.

Rg3‐induced GFP::OSBP(PH)‐positive structures partially overlapped with LysoTracker Red‐positive acidic compartments, linking PI(4)P remodeling to lysosome‐associated membranes (Figure 5F,G). We then tested *pifk‐1* and *vps‐34*, associated with PI(4)P and PI(3)P production, respectively (Ebner et al. 2023) (Figure 5H). Rg3 reduced Q40 aggregation under control RNAi but not *pifk‐1* RNAi (Figure 5I). Its locomotor benefit was also absent after *pifk‐1* or *vps‐34* knockdown (Figure 5J), implicating these phosphoinositide‐regulatory genes in its functional effects.

Together, these findings associate Rg3 treatment with altered neuronal phosphoinositide reporter distributions and PIFK‐1‐dependent proteostatic and behavioral benefits.

### 3.6 Rg3 Engages PIFK‐1‐Dependent Phosphoinositide Remodeling Coupled to TORC1 Suppression and HLH‐30 Activation

Transcriptomic analysis showed reduced *let‐363*/TOR and *daf‐15*/Raptor expression and increased *pifk‐1* expression following Rg3 treatment (Figure 6A and Figure S5A,B). LET‐363 and DAF‐15 are core TORC1 components (Ebner et al. 2023). Rg3 also reduced RSKS‐1/S6K phosphorylation (Figure 6B,C), consistent with lower TORC1‐associated signaling. Additional analyses further supported the involvement of TORC1 signaling, as Rg3 preferentially reduced the expression of TORC1‐associated genes relative to TORC2‐associated genes (Figure S5C,D).

**FIGURE 6:**
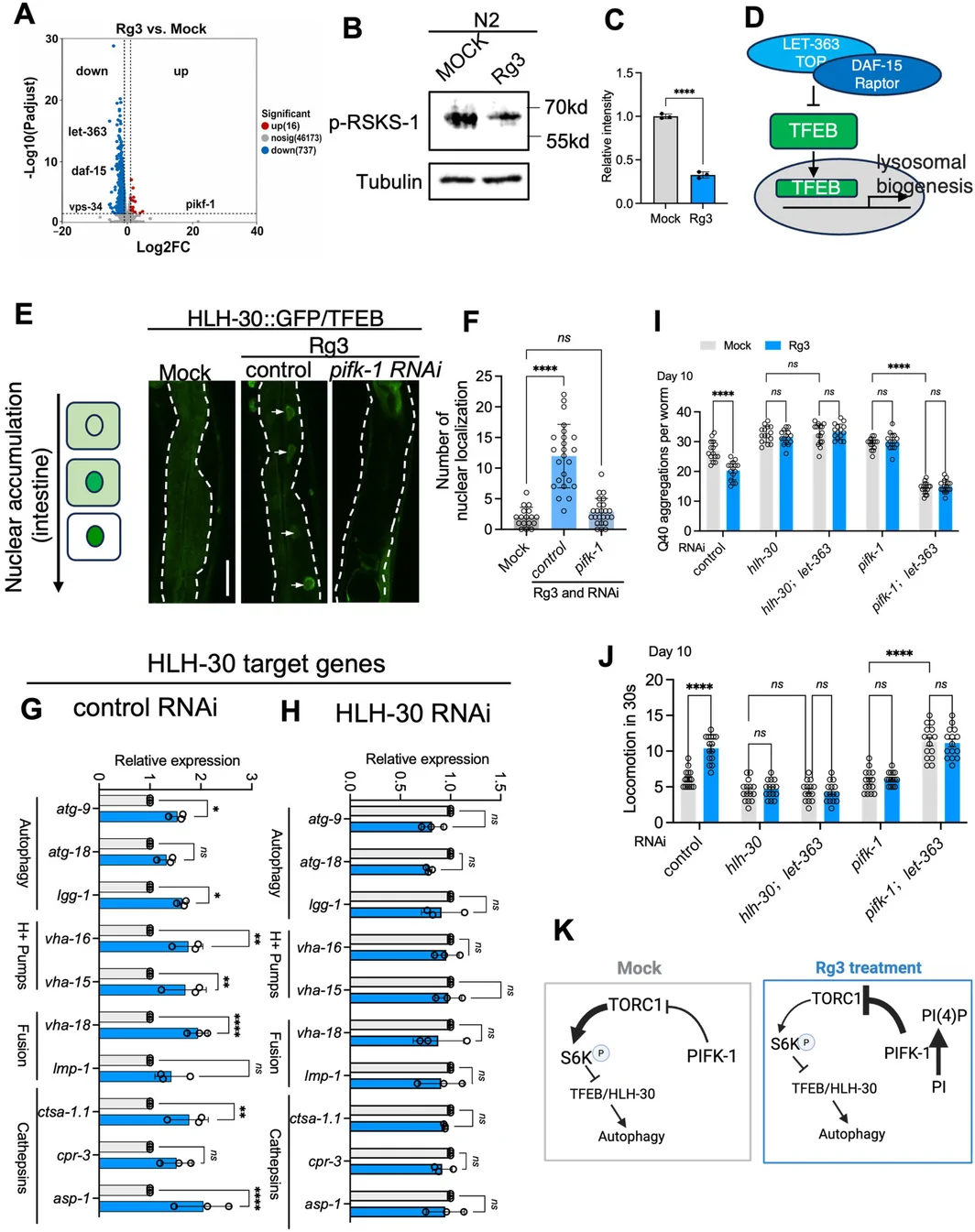
Rg3 suppresses RSKS‐1 phosphorylation and activates a PIFK‐1‐ and HLH‐30‐dependent lysosomal response. (A) Volcano plot of differentially expressed genes in Rg3‐treated animals relative to vehicle‐treated controls. Vertical and horizontal dashed lines indicate |log_2_(fold change)| = 1 and adjusted *p* = 0.05, respectively. Selected genes associated with TOR signaling and phosphoinositide metabolism, including *let‐363*, *daf‐15*, *vps‐34*, and *pifk‐1*, are indicated. (B, C) Representative immunoblot (B) and densitometric quantification (C) of phosphorylated RSKS‐1/S6K in vehicle‐ and Rg3‐treated animals. Phosphorylated RSKS‐1 abundance was normalized to α‐tubulin. (D) Schematic illustrating the conserved regulation of TFEB/HLH‐30 by TORC1. (E, F) Representative images (E) and quantification (F) of HLH‐30::GFP nuclear localization following vehicle treatment, Rg3 treatment, or combined Rg3 treatment and *pifk‐1* RNAi. Scale bar, 40 μm. (G, H) Reverse‐transcription quantitative PCR analysis of the indicated HLH‐30 target genes associated with autophagy, proton transport, vesicle fusion, and lysosomal proteolysis in animals subjected to control or *hlh‐30* RNAi and treated with vehicle or Rg3. Expression was normalized to *cdc‐42* and is shown relative to vehicle‐treated control‐RNAi animals. (I, J) Effects of Rg3 on (I) neuronal Q40::YFP aggregation and (J) locomotor activity on adult Day 10 following control, *hlh‐30*, *hlh‐30; let‐363*, *pifk‐1*, or *pifk‐1; let‐363* RNAi. (K) Proposed working model in which Rg3 promotes PIFK‐1‐associated PI(4)P remodeling, attenuates TORC1–RSKS‐1 signaling, activates HLH‐30‐dependent autophagy–lysosome gene expression, and thereby preserves neuronal proteostasis during aging. The diagram was created using BioRender.com. Data are mean ± SD from three independent biological experiments. For immunoblotting and RT–qPCR, *n* = 3 independent biological replicates. For imaging and behavioral assays, *n* ≥ 15 animals per condition from three independent experiments. Data were analyzed using unpaired two‐tailed *t*‐test, one‐way ANOVA, or two‐way ANOVA, followed by Šídák's multiple‐comparison test. *p < 0.05, **p < 0.01, ****p < 0.0001; ns, not significant.

Rg3 promoted nuclear accumulation of HLH‐30::GFP, whereas *pifk‐1* RNAi abolished this response (Figure 6D–F). Thus, PIFK‐1‐dependent signaling is required for Rg3‐induced HLH‐30 nuclear localization and may act upstream of, or be functionally coupled to, HLH‐30 activation.

Rg3 increased expression of autophagy–lysosome genes, including *atg‐9*, *lgg‐1*, *vha‐16*, *vha‐15*, *vha‐18*, *ctsa‐1.1*, and *asp‐1*, under control RNAi (Figure 6G). These responses were largely diminished or abolished following *hlh‐30* RNAi (Figure 6H), supporting HLH‐30‐dependent transcriptional regulation.

HLH‐30 was also required for the functional response: Rg3 reduced neuronal Q40 aggregation and improved locomotion on adult Day 10 under control RNAi, but these benefits were lost following *hlh‐30* knockdown (Figure 6I,J).

Finally, simultaneous *let‐363* knockdown restored protection in the *pifk‐1* RNAi background, reducing Q40 aggregation and improving locomotion (Figure 6I,J). Rg3 did not confer further detectable protection in this double‐knockdown background. These findings suggest that TORC1 suppression can bypass, at least partly, the requirement for PIFK‐1‐dependent signaling.

Together, these findings suggest that Rg3 promotes PIFK‐1‐dependent phosphoinositide remodeling that is functionally coupled to TORC1–RSKS‐1 suppression and HLH‐30 activation (Figure 6K). Through this pathway, Rg3 induces a lysosomal‐autophagy transcriptional program, reduces neuronal proteotoxic stress, ameliorates motor aging, and supports organismal healthspan (Figure 6K).

## 4 Discussion

This study identifies 20(S)‐ginsenoside Rg3 as an adult‐active intervention that extends lifespan and preserves healthspan traits in *C. elegans* . Benefits persisted with heat‐killed bacteria and adult‐onset treatment and were not accompanied by reduced progeny production. Rg3 also preserved neuronal morphology and sensory behavior, reduced Q40 aggregation, and protected dopaminergic neurons in an α‐synuclein model.

Our findings connect a natural compound with phosphoinositide‐associated lysosomal regulation during aging. Rg3 increased the PI(4)P‐to‐PI(3)P reporter index, reduced RSKS‐1 phosphorylation, and promoted HLH‐30 nuclear accumulation. Nutrient‐regulated phosphoinositide conversion has been linked to lysosomal function and mTORC1–TFEB signaling (Ebner et al. 2023). Our results are consistent with a related signaling response in Rg3‐treated worms, although reporter measurements do not establish absolute lipid concentrations or direct conversion between phosphoinositides.

Partial VPS‐34 inhibition can ameliorate motor aging through changes in PI(3)P/PI(4)P regulation (Hu et al. 2023). Our results extend this framework by associating Rg3 treatment with altered phosphoinositide reporter behavior and PIFK‐1‐dependent protection. Genetic suppression experiments further support a functional relationship between PIFK‐1 and TORC1, while leaving the precise sequence and cellular localization of these events unresolved.

By studying a defined ginsenoside rather than a complex ginseng extract, we identify a specific natural product with organismal and neuronal benefits. The connection between Rg3, lysosomal function, and proteostasis may be relevant to age‐associated neurodegenerative conditions, but efficacy and mechanism in mammalian systems remain to be established.

The direct molecular target of Rg3 remains unknown. Future studies should test whether Rg3 directly regulates PIFK‐1 or instead acts through metabolic or trafficking pathways. Defining the tissue‐specific requirements for PIFK‐1, TORC1, and HLH‐30 will clarify how neuronal and intestinal responses coordinate organismal healthspan. Mammalian studies are also needed to assess whether lysosomal PI(4)P regulation and TFEB activation mediate comparable responses.

In summary, Rg3 extends lifespan and preserves neuronal health in *C. elegans* . The findings support a model in which phosphoinositide‐associated lysosomal regulation is functionally coupled to TORC1–RSKS‐1 suppression and HLH‐30 activation, providing a framework for further investigation of natural‐product interventions in aging.

## Author Contributions

Haiyang Yu, Shiming Li, and Yongsheng Chen performed the experiments and analyzed the data. Yaoyong Zeng, Junbu Li, Yuxin Guo, Wanxiang Zhang, Lizhu Zhang, Longnong You, Kuncan Chen, and Jinhong Wu assisted with the experiments and data collection. Shiming Li, Jiane‐Kang Liu, and Lingjun Zheng drafted and revised the manuscript. Dan Gan, Jiane‐Kang Liu, and Lingjun Zheng conceived and designed the study and supervised the project.

## Funding

This work was supported by the National Natural Science Foundation of China (NSFC; grant nos. 32470877, 82401817, and 82603057); the Shanghai Oriental Talent Award (grant no. QNJY2024184); the Shanghai Natural Science Foundation (grant no. 24ZR1440500); the Science and Technology Commission of Shanghai Municipality (grant no. 26010700900); the Ningbo Science and Technology Bureau through the Kechuang Yongjiang 2035 Ningbo Key Research and Development Plan (grant no. 2024Z280); and the Shanghai Municipal Agriculture and Rural Affairs Commission (grant no. V2025012). Additional financial support was provided by Sirio Pharma Co. Ltd.

## Conflicts of Interest

D.G. and W.Z. are employees of Sirio Pharma Co. Ltd. The remaining authors declare that they have no known competing financial interests or personal relationships that could have appeared to influence the work reported in this paper.

## Supporting information


**Figure S1:** Rh2 does not phenocopy Rg3, and Rg3‐mediated lifespan extension is not explained by inhibition of bacterial growth or food avoidance. (A) Chemical structures of 20(S)‐ginsenoside Rg3 and its metabolite Rh2. (B) Survival curves of wild‐type N2 animals treated with vehicle (Mock) and Rh2. (C) Growth curves of *Escherichia coli* OP50 cultured in the presence of vehicle or Rg3, as determined by optical density at 600 nm (OD₆₀₀). (D) Two‐choice food preference assay in which animals were allowed to choose between vehicle‐ and Rg3‐supplemented OP50 lawns. Food preference was expressed as percentage of animals in each bacterial lawn. For bacterial growth and food preference assays, data are mean ± SD from three independent experiments.
**Figure S2:** Rg3 preserves touch receptor and chemosensory neuron morphology and function during aging. (A) Representative images of anterior lateral microtubule (ALM) and posterior lateral microtubule (PLM) touch receptor neurons labeled with *mec‐4p::GFP* in vehicle‐ and Rg3‐treated animals on adult Days 3, 5 and 10. Scale bar, 20 μm. (B) Distribution of ALM morphological categories (well‐organized, moderately disorganized, or severely disorganized). (C) Distribution of PLM morphological categories, as defined in (B). (D) Representative images of AWC^ON^ chemosensory neurons labeled with *str‐2p::GFP* in vehicle‐ and Rg3‐treated animals at the indicated adult ages. Scale bar, 20 μm. (E) Distribution of AWC morphological categories. Neuronal morphology was scored according to the predefined criteria described in the Materials and methods by an investigator blinded to treatment. (F) Schematic of the chemotaxis assay. The chemotaxis index was calculated as CI = (*N*odorant − *N*control)/(*N*odorant + *N*control), where *N*odorant and *N*control denote the numbers of animals in the odorant and vehicle‐control regions, respectively. (G, H) Chemotaxis indices toward (G) isoamyl alcohol (isopentanol) and (H) benzaldehyde in vehicle‐ and Rg3‐treated animals on adult Day 3. For morphological analyses, data represent the percentages of animals in each predefined category, based on three independent experiments with *n* ≥ 15 animals per condition. Morphological category distributions were compared between Mock and Rg3 groups at each age using two‐sided Fisher's exact tests. Chemotaxis indices are presented as mean ± SD from three independent experiments and were analyzed using two‐way ANOVA followed by Šídák's multiple‐comparison test. *p < 0.05, **p < 0.01, ****p < 0.0001; ns, not significant.
**Figure S3:** Rg3 increases the mCherry‐to‐GFP fluorescence ratio of a neuronal tandem LGG‐1 reporter during aging. (A) Representative images of the neuron‐specific tandem fluorescent autophagy reporter *rgef‐1p::mCherry::GFP::lgg‐1* in animals treated with vehicle or Rg3 on adult Days 3, 5 and 10. Dotted lines indicate the body outline. Scale bar, 20 μm. (B) Quantification of the neuronal mCherry‐to‐GFP fluorescence ratio. Rg3 did not alter the reporter ratio on adult Day 3 but increased it on Days 5 and 10, consistent with increased accumulation of LGG‐1 in acidic autolysosomal compartments. Data are mean ± SD; each point represents one animal. Data were obtained from three independent experiments, with *n* ≥ 15 animals per condition. Statistical comparisons were performed using two‐way ANOVA followed by Šídák's multiple‐comparison test. *p < 0.05, **p < 0.01, ***p < 0.001, ****p < 0.0001; ns, not significant.
**Figure S4:** Rg3 preserves lysosomal morphology and alters fluorescence‐based indices of lysosomal acidity during aging.
(A) Representative images of hypodermal lysosomes labeled with NUC‐1::mCherry in vehicle‐ and Rg3‐treated animals on adult Days 3, 5 and 10. Scale bars, 20 μm. (B) Quantification of lysosomal tubule length at the indicated adult ages. (C) Quantification of vesicular lysosome accumulation per animal. (D) Representative images of intestinal LysoSensor Green and LysoTracker Red staining in vehicle‐ and Rg3‐treated animals at the indicated adult ages. Scale bar, 20 μm. (E) Quantification of the relative lysosomal acidity index, calculated as the normalized LysoSensor Green‐to‐LysoTracker Red fluorescence ratio. Data are mean ± SD from three independent experiments, with *n* ≥ 15 animals per condition. Data were analyzed using two‐way ANOVA followed by Šídák's multiple‐comparison test. *p < 0.05, **p < 0.01, ***p < 0.001, ****p < 0.0001; ns, not significant.
**Figure S5:** Rg3 alters the expression of TORC1‐associated and phosphoinositide‐regulatory genes and supports a phosphoinositide–TORC1–HLH‐30 working model. (A) Heatmap showing the expression of selected age‐ and Rg3‐responsive genes in Day‐1 adults (D1) and Day‐5 adults treated with vehicle (D5 Mock) or Rg3 (D5 Rg3). Each column represents an independent biological replicate. Expression values were row‐normalized and are displayed as *Z*‐scores. (B) Reverse‐transcription quantitative PCR validation of *let‐363*, *daf‐15*, *rict‐1* and *pifk‐1* expression in Day‐5 adults treated with vehicle or Rg3. Expression was normalized to *cdc‐42* and is shown relative to the corresponding vehicle‐treated group. (C) Schematic of the *C. elegans* TOR complexes. TORC1 contains LET‐363/TOR and DAF‐15/Raptor, whereas TORC2 contains LET‐363/TOR and RICT‐1/Rictor. LET‐363 is the shared catalytic kinase of both complexes. (D) Summary of Rg3‐induced changes in the expression of TORC1‐ and TORC2‐associated components, calculated relative to vehicle‐treated controls. The shared component *let‐363* is displayed in both groups to illustrate its participation in both complexes and should not be interpreted as two independent measurements. (E) Proposed working model. Rg3 shifts the neuronal phosphoinositide reporter balance toward PI(4)P, attenuates TORC1‐associated signaling and promotes activation of HLH‐30/TFEB. Increased HLH‐30 activity is proposed to enhance autophagy–lysosome gene expression and lysosomal function, thereby preserving neuronal proteostasis and supporting healthy lifespan. For RT–qPCR, data are mean ± SD from three independent biological experiments. Vehicle‐ and Rg3‐treated groups were compared using multiple unpaired two‐tailed t‐tests with Holm–Šídák correction. *p < 0.05, ****p < 0.0001; ns, not significant.

**Table S1:** acel70755‐sup‐0002‐TableS1‐S5.xlsx. *C. elegans* strains and transgenes used in this study.
**Table S2:** Oligonucleotide primers used in this study.
**Table S3:** RNAi feeding reagents mentioned in the Rg3 manuscript.
**Table S4:** Axon regeneration genes.
**Table S5:** FYVE‐domain‐containing genes in the current C. elegans DEG screen.

## Data Availability

The RNA‐seq datasets have been deposited in the NCBI Gene Expression Omnibus under accession number GSE339194 (https://www.ncbi.nlm.nih.gov/geo/query/acc.cgi?acc=GSE339194). Additional data supporting this study are available from the corresponding authors upon reasonable request. Worm strains used or generated in this study are available from the Caenorhabditis Genetics Center or the authors, as applicable.
