# Supplementary material for: Ginsenoside Rg3 Promotes Healthy Aging Through Phosphoinositide‐Associated Lysosomal Regulation in Caenorhabditis elegans
Source: Aging Cell. 2026 Oct 10;25(10):e70755. doi: 10.1111/acel.70755 (PMC13653540; doi:10.1111/acel.70755)
Supplement: Supplementary file 1 [file ACEL-25-e70755-s001.docx]

### **Supporting Information**

 **Figure S1. Rh2 does not phenocopy Rg3, and Rg3-mediated lifespan extension is not explained by inhibition of bacterial growth or food avoidance.**
(A) Chemical structures of 20(S)-ginsenoside Rg3 and its metabolite Rh2. (B) Survival curves of wild-type N2 animals treated with vehicle (Mock) and Rh2. (C) Growth curves of Escherichia coli OP50 cultured in the presence of vehicle or Rg3, as determined by optical density at 600 nm (OD₆₀₀). (D) Two-choice food preference assay in which animals were allowed to choose between vehicle- and Rg3-supplemented OP50 lawns. Food preference was expressed as percentage of animals in each bacterial lawn. For bacterial growth and food preference assays, data are mean ± SD from three independent experiments.

**Figure S2. Rg3 preserves touch receptor and chemosensory neuron morphology and function during aging.**
(A) Representative images of anterior lateral microtubule (ALM) and posterior lateral microtubule (PLM) touch receptor neurons labeled with mec-4p::GFP in vehicle- and Rg3-treated animals on adult days 3, 5 and 10. Scale bar, 20 μm. (B) Distribution of ALM morphological categories (well-organized, moderately disorganized, or severely disorganized). (C) Distribution of PLM morphological categories, as defined in (B). (D) Representative images of AWC^ON^ chemosensory neurons labeled with str-2p::GFP in vehicle- and Rg3-treated animals at the indicated adult ages. Scale bar, 20 μm. (E) Distribution of AWC morphological categories. Neuronal morphology was scored according to the predefined criteria described in the Materials and methods by an investigator blinded to treatment. (F) Schematic of the chemotaxis assay. The chemotaxis index was calculated as CI = (Nodorant − Ncontrol)/(Nodorant + Ncontrol), where Nodorant and Ncontrol denote the numbers of animals in the odorant and vehicle-control regions, respectively. (G,H) Chemotaxis indices toward (G) isoamyl alcohol (isopentanol) and (H) benzaldehyde in vehicle- and Rg3-treated animals on adult day 3.

For morphological analyses, data represent the percentages of animals in each predefined category, based on three independent experiments with n ≥ 15 animals per condition. Morphological category distributions were compared between Mock and Rg3 groups at each age using two-sided Fisher’s exact tests. Chemotaxis indices are presented as mean ± SD from three independent experiments and were analyzed using two-way ANOVA followed by Šídák’s multiple-comparison test. *P < 0.05, **P < 0.01, ****P < 0.0001; ns, not significant.

**Figure S3. Rg3 increases the mCherry-to-GFP fluorescence ratio of a neuronal tandem LGG-1 reporter during aging.**
(A) Representative images of the neuron-specific tandem fluorescent autophagy reporter rgef-1p::mCherry::GFP::lgg-1 in animals treated with vehicle or Rg3 on adult days 3, 5 and 10. Dotted lines indicate the body outline. Scale bar, 20 μm. (B) Quantification of the neuronal mCherry-to-GFP fluorescence ratio. Rg3 did not alter the reporter ratio on adult day 3 but increased it on days 5 and 10, consistent with increased accumulation of LGG-1 in acidic autolysosomal compartments.

Data are mean ± SD; each point represents one animal. Data were obtained from three independent experiments, with n ≥ 15 animals per condition. Statistical comparisons were performed using two-way ANOVA followed by Šídák’s multiple-comparison test. *P < 0.05, **P < 0.01, ***P < 0.001, ****P < 0.0001; ns, not significant.

**Figure S4. Rg3 preserves lysosomal morphology and alters fluorescence-based indices of lysosomal acidity during aging.**
(A) Representative images of hypodermal lysosomes labeled with NUC-1::mCherry in vehicle- and Rg3-treated animals on adult days 3, 5 and 10. Scale bars, 20 μm. (B) Quantification of lysosomal tubule length at the indicated adult ages. (C) Quantification of vesicular lysosome accumulation per animal. (D) Representative images of intestinal LysoSensor Green and LysoTracker Red staining in vehicle- and Rg3-treated animals at the indicated adult ages. Scale bar, 20 μm. (E) Quantification of the relative lysosomal acidity index, calculated as the normalized LysoSensor Green-to-LysoTracker Red fluorescence ratio.

Data are mean ± SD from three independent experiments, with n ≥ 15 animals per condition. Data were analyzed using two-way ANOVA followed by Šídák’s multiple-comparison test. *P < 0.05, **P < 0.01, ***P < 0.001, ****P < 0.0001; ns, not significant.

**Figure S5. Rg3 alters the expression of TORC1-associated and phosphoinositide-regulatory genes and supports a phosphoinositide–TORC1–HLH-30 working model.**
(A) Heatmap showing the expression of selected age- and Rg3-responsive genes in day-1 adults (D1) and day-5 adults treated with vehicle (D5 Mock) or Rg3 (D5 Rg3). Each column represents an independent biological replicate. Expression values were row-normalized and are displayed as Z-scores. (B) Reverse-transcription quantitative PCR validation of let-363, daf-15, rict-1 and pifk-1 expression in day-5 adults treated with vehicle or Rg3. Expression was normalized to *cdc-42* and is shown relative to the corresponding vehicle-treated group. (C) Schematic of the C. elegans TOR complexes. TORC1 contains LET-363/TOR and DAF-15/Raptor, whereas TORC2 contains LET-363/TOR and RICT-1/Rictor. LET-363 is the shared catalytic kinase of both complexes. (D) Summary of Rg3-induced changes in the expression of TORC1- and TORC2-associated components, calculated relative to vehicle-treated controls. The shared component let-363 is displayed in both groups to illustrate its participation in both complexes and should not be interpreted as two independent measurements. (E) Proposed working model. Rg3 shifts the neuronal phosphoinositide reporter balance toward PI(4)P, attenuates TORC1-associated signaling and promotes activation of HLH-30/TFEB. Increased HLH-30 activity is proposed to enhance autophagy–lysosome gene expression and lysosomal function, thereby preserving neuronal proteostasis and supporting healthy lifespan.

For RT–qPCR, data are mean ± SD from three independent biological experiments. Vehicle- and Rg3-treated groups were compared using multiple unpaired two-tailed t-tests with Holm–Šídák correction. *P < 0.05, ****P < 0.0001; ns, not significant.
